# Dimensional Latent Structure of Callous-Unemotional Traits in German Adolescents: Results from Taxometric Analyses

**DOI:** 10.1007/s10802-021-00885-y

**Published:** 2021-11-13

**Authors:** Sören Kliem, Yvonne Krieg, Thimna Klatt, Dirk Baier

**Affiliations:** 1 Ernst-Abbe-Hochschule Jena – University of Applied Sciences, Carl-Zeiss-Promenade 2, 07745 Jena, Germany; 2 Criminological Research Institute of Lower Saxony, Hannover, Germany; 3 University of Applied Sciences for Police and Public Administration in North Rhine-Westphalia, Hagen, Germany; 4 Zurich University of Applied Sciences, Zürich, Switzerland

**Keywords:** Callous Unemotional Traits (CU traits), Psychopathy, Taxometrics, Latent Structure, Assessment

## Abstract

A large amount of research has addressed the issue of the latent status of psychiatric disorders and related phenomena. We used a new taxometric approach developed by Ruscio to examine the latent status of callous-unemotional (CU) traits in a large representative study of German ninth graders (*N* = 3,878). Rather than estimating a putative taxon base rate and using that estimate to generate the taxon comparative data, we estimated CCFI profiles with each base rate estimate between 2.5% and 97.5% in increments of 2.5%. Results of different indicator sets clearly suggested a dimensional solution. This finding is consistent with different studies showing the dimensionality of psychopathy in adolescents. In summary, the results of this study point to the need for critical reflection in defining a high-risk-group in the context of CU traits. However, further studies are necessary to substantiate this result in different samples using different measurement approaches.

Callous-unemotional (CU) traits have been found to be closely related to the affective dimension of psychopathy (Hare & Neumann, 2008; Kimonis et al., 2015). Different authors argue that CU traits are useful for identifying a high-risk group (CU +) within children with Conduct Disorder (CD; Frick & White, 2008). This group is characterized by marked differences in neurocognitive, emotional, and behavioral functioning, including lower autonomic responsiveness to empathy-inducing stimuli (Frick, & Viding, 2009; de Wied et al., 2012), disturbances in affective theory of mind (Sebastian et al., 2012), lower sensitivity to punishment (Frick et al., 2014), and changes in brain regions involved in emotion and learning (e.g., amygdala, Blair et al., 2014). Collectively, these characteristics are assumed to contribute to the more violent, chronic, and recidivist pattern of antisocial behavior exhibited by youth with high CU traits and are an important target for intervention (Cecil et al., 2018). Furthermore, it has been demonstrated that treatment non-responders have significantly higher CU levels than responders (e.g., Falkenbach et al., 2003; Gretton et al., 2001; Hawes & Dadds, 2005; Masi et al., 2013; O'Neill et al., 2003; Spain et al., 2004; Waschbusch et al., 2007). Moreover, CU traits in school-aged children predict later criminal and antisocial behavior in adulthood, even after controlling for CD severity and onset (McMahon et al., 2010).

For these reasons, the DSM-5 revisions (American Psychiatric Association, 2013) added the possibility of additional coding (descriptive features specifier) "with Limited Prosocial Emotions" (LPE) to the CD diagnosis. To warrant this additional coding, at least two of the four specifiers must occur within the same time period and across different relationships and situations: (a) lack of remorse or guilt; (b) callous lack of empathy; (c) unconcerned about performance; (d) shallow or deficient affect. These four criteria closely approximate the affective dimension of psychopathy in adult samples (Hare & Neumann, 2008). Accordingly, the DSM-5 follows a categorical conceptualization (with specifier [high risk] vs. without specifier) of CU traits.

## Latent Structure of CU Traits

In addition to conceptualizing CU traits as categorical (i.e., identifying high-risk individuals who score above a certain cut-off value), they can also be understood as forming a latent continuum. A number of studies have been conducted to investigate if certain psychiatric disorders consist of discrete categories of behaviors, or if they rather form a continuum connecting extreme forms of behavioral traits on a single dimension (Haslam et al., 2020). The issue of whether a phenomenon (e.g., a mental disorder) is appropriately conceptualized as "dimensional" (i.e., as manifestations along a continuum of behavioral characteristics) or as discrete categorical entities has important implications for research, theory, and practice (Ruscio & Ruscio, 2004). For example, the latent status of a construct is important for the classification of individuals. If the underlying construct is continuous, the convention for classification into dichotomous groups (e.g., treatment vs. no treatment) must be derived based on certain criteria that are not part of the diagnosis (external validation criteria). If, on the other hand, a true categorical latent structure is present, providing clinically relevant cut-off values to differentiate the corresponding groups appears to be an essential target. Furthermore, identifying the latent structure of a phenomenon is also important to guide research into its etiopathogenesis (i.e., the cause and development of an atypical condition or disease). It can be argued that a dimensional structure is rather generated by a multitude of different risk factors through addition and interaction. On the other hand, existence of categorical latent structure can result from a specific etiology or developmental bifurcation (see Meehl, 1995; Ruscio et al., 2006). Moreover, in the context of prognostic studies (i.e., using CU traits as an explanatory factor) or studies on etiological factors (i.e., CU traits as the outcome), the latent status of a phenomenon seems to be of particular importance and should influence the selection of the appropriate statistical procedures.

To determine whether the latent structure of a construct is best conceptualized as dimensional or categorical, taxometric methods are often used. Taxometric techniques were originally discussed by Paul E. Meehl to test his conjecture that a discrete latent variable ("taxon") underlies vulnerability to schizophrenia (Golden & Meehl, 1979). Meehl (1995) introduced a fundamental feature into modern taxometric analyses. Several nonredundant data-analytic procedures (see Ruscio et al., 2011 for a detailed description) are applied and the final interpretation of the latent structure of the construct are based on the convergence among these procedures.

A significant methodological development in taxometric analysis represents the introduction of a systematized approach to taxometric inference by Ruscio et al. (2007). These authors developed a procedure in which taxometric plots based on observed data are compared with plots from parallel analyses of matched (e.g., sample size, marginal distributions, correlation matrix) simulated comparison datasets generated from a population of data using a taxonomic or dimensional latent structural model. In addition, the authors developed an index (Comparison Curve Fit Index, CCFI) which quantifies the similarity of the observed curves from the simulated curves. A CCFI value < 0.45 indicates a dimensional structure, a CCFI value of > 0.55 indicates a categorical structure. Values between 0.45 and 0.55 are considered ambiguous. The CCFI value can be calculated independently for the different taxometric procedures. A final interpretation is then usually based on a mean CCFI value (Ruscio et al., 2018). This method of simulated comparative data set and the use of CCFI have become almost universally accepted (Haslam et al., 2020).

A number of previous taxometric studies consistently support the dimensionality of psychopathy in adolescents (Edens et al., 2011; Murrie et al., 2007; Vasey et al., 2005; Walters, 2014). However, to the authors’ best knowledge only one study has examined the latent structure of CU traits so far. Herpers et al. (2017) analyzed the data of *N* = 979 Dutch children and adolescents using taxometric analysis. The results of their study, namely the Comparison Curve Fit Index (CCFI; Ruscio et al., 2007), point to a dimensional latent structure of CU traits. However, a number of limitations apply to the Herpers et al. study. The authors did not provide any information on what type of indicator they used and whether the requirements for the analysis were met (i.e., within-group correlations, indicator validity, number of indicators, number of ordered categories). In addition, the estimated baseline prevalence of the possible taxon subgroup, the estimation method and subgroup analyses (e. g. regarding gender) were not provided. In the present study, we replicate the taxometric analysis of CU traits, avoiding the limitations of the study by Herpers and colleagues. Data for this analysis were obtained from a representative sample of ninth graders in Germany. Following recent developments in the methodology of taxometric analysis, we will use a new taxometric approach developed by Ruscio et al. (2018), the CCFI profile method. Rather than estimating a putative taxon base rate and using that estimate to generate the taxon comparative data, the CCFI profile method replicates the analysis with each base rate estimate between 0.025 and 0.975 in increments of 0.025.

## Method

### Sampling Method

The following analysis uses child-report data from ninth grade students in Germany originating from a periodically conducted representative survey (see Kliem et al., 2020), carried out by the Criminological Research Institute of Lower Saxony in spring 2015. The Ministry of Education of Lower Saxony (this constitutes the state’s educational authority) approved the survey and provided ethics auditing. The survey was strictly anonymized – neither names, nor private or school addresses were obtained. The study was conducted in accordance with the World Medical Association’s (WMA) Declaration of Helsinki. The survey was carried out by trained test administrators within a classroom setting and was completed in a time frame of two school lessons (90 min). The students’ parents received an information leaflet beforehand, which included a request for written consent for the participation of their child and provided them with information about the institution conducting the study, as well as aims, methods and financing of the study. Furthermore, the students themselves could also independently refuse to participate, despite the existing consent given by their parents. Students were informed that their participation in the survey is entirely voluntary and anonymous and that they could withdraw their participation consent at any time without any negative consequences. Furthermore, they were informed of their right to skip individual questions within the survey and were encouraged to speak to a counsellor, school psychologist or an anonymous crisis hotline if they were to feel negatively affected by partaking in the survey. Of the *N* = 3,878 students who participated, 51.4% are female (*n* = 1,992 individuals). The mean age is *M* = 14.9 years (*SD* = 0.71), with an age range of 13 to 19 years. *N* = 926 (23.9%) of the respondents have a migration background (i.e., students or at least one of their parents were not born in Germany or do not have German citizenship).

### Measures

The *Inventory of Callous-Unemotional Traits* (ICU) by Frick (2004) can be considered the current standard for assessing CU traits (e. g. Cardinale & Marsh, 2020; Frick & Ray, 2015; Frick et al., 2014; Ray & Frick, 2020). The ICU is based on four items of the CU scale of Frick and Hare’s *Antisocial Process Screening Device* (APSD; Frick & Hare, 2001). These four original APSD items formed the basis of the four subscales Uncaring, Unemotional, Callous and Careless. These subscales correspond to the LPE dimensions of the DSM-5 (see Kimonis et al., 2015). A German version of the ICU (Frick, 2004; German version by Essau et al., 2006) was used to record child-reported insensitive, insidious, and hard-hearted properties. On the ICU, the young people indicate how accurately each item describes their own behavior (from 0 = "not at all true" to 3 = "definitely true").

### Statistical Methods

#### Missing Values

Missing values (all included items < 5% missing data) were estimated using Chained Equation Modelling (see White et al., 2011). To avoid the imputation of item values, which do not correspond to the possible characteristics of the items, estimated values are in turn replaced by the “nearest natural neighbor” (Predictive Mean Matching Method, Little, 1988). Imputation was carried out using the R package *mice* (Multivariate Imputation by Chained Equations in R; van Buuren & Groothuis-Oudshoorn, 2011).

#### Indicator Selection

We tested two different three-indicator sets based on the work of Essau et al. (2006) [*Uncaring* (#3, #5, #13, #15, #16, #17, #23, #24), *Unemotional* (#1, #6, #14, #19, #22) *Callous* (#2, #4, #7, #8, #9, #10, #11, #12, #18, #20, #21)] and Kimonis et al. (2015) (excluding item #2 and #10). Furthermore, we analyzed two four-indicator sets on the original model of the APSD [*Uncaring* (#4, #8, #12, #17, #21, #24), *Unemotional* (#1, #6, #10, #14, #19, #22), *Callous* (#2, #5, #9, #13, #16, #18), and *Careless* (#3, #7, #11, #15, #20, #23)] and the work of Kliem et al. (2020) (excluding item #2, #10, and #13).

#### Taxometric Analysis

As recommended by Ruscio et al. (2010), we applied three non-redundant taxometric procedures: Mean above minus below a cut (MAMBAC Meehl & Yonce, 1994;), maximum eigenvalue (MAXEIG; Waller & Meehl, 1998), and latent-mode factor analysis (L-MODE; Waller & Meehl, 1998). Following the suggestion by Ruscio et al., (2007; see Ruscio et al. (2011) for a comprehensive introduction), two comparison populations (each *N* = 100,000) using (a) the categorical model and (b) the dimensional model were generated for each of the taxometric procedures. Relevant aspects of the empirical data, such as skewness, inter-correlations, and non-normality were held constant. In a second step, random samples (*K* = 100; with the same sample size of the empirical data set) were drawn from both populations. The R package *RTaxometrics* by Ruscio and Wang (2017) was used for these simulations. All samples were then analyzed using the three different taxometric procedures (MAMBAC, MAXEIG, L-MODE).

The root-mean-square distance between empirical data points on curves and data points on simulated categorical (FitCat) as well as simulated dimensional (FitDim) reference curves were calculated (smaller values indicating that both curves resemble one another more closely). Next, the comparison curve fit index (CCFI = FitDim / (FitDim + FitCat)) was calculated for each taxometric procedure. In accordance with Ruscio et al. (2010), the mean CCFI of the MAMBAC, MAXEIG, and L-MODE procedure was used to interpret the latent status of CU traits. Rather than estimating a putative taxon base rate and using that estimate to generate the taxon comparative data, we used the CCFI profile method developed by Ruscio et al. (2018). This method replicates the analysis with each base rate estimate between 2.5% and 97.5% in increments of 2.5%. If the construct is taxonic, the CCFI value should be greatest at the most accurate base rate estimation (Ruscio et al., 2018). In Monte Carlo simulations, this method provided a more accurate base rate estimation (in the case of categorical structure) as well as a particularly adequate estimate of latent structure on the basis of a CCFI profile value, whereby a CCFI profile value above 0.50 denotes a better fit for a categorical latent structure and a value below 0.50 denotes a better fit for a dimensional latent structure (Ruscio et al., 2018). We used Ruscio’s and Wang’s R package *RTaxometrics* (Ruscio & Wang, 2017) for the analysis. We performed CCFI profile analyses for the total sample as well as for males and females separately.

#### Suitability of Data for Taxometric Analysis

To check the prerequisites for taxometric analysis, assigning cases to putative groups is necessary. Based on Ruscio's, Ruscio's, and Carney's recommendations, case classification should be based on a meaningful diagnostic algorithm or valid assessment tool. It should be noted that any of these classification procedures is necessarily based on the assumption of a categorical latent structure. If taxometric results indicate a dimensional structure, this classification must however be questioned. Also, the determined base rates (see below) should then not be interpreted further. We used a group variable (taxon vs. complement) based on an algorithm presented by Kimonis et al. (2015). Four CU items (#3, #5, #6, and #8) were dichotomized (coded as present if rated 3 “definitely true”; see Kimonis et al., 2015). The following two groups were formed: Those reporting no symptoms or one symptom (i.e., not meeting CU specifier criteria) and those reporting ≥ 2 symptoms (i.e., meeting specifier criteria), reflecting the DSM-5 symptom threshold (APA, 2013). Based on this threshold, we found a base rate for the putative taxon group of 8.1% (n = 313) for the total sample, of 10.9% (*n* = 205) for the male sample as well as of 5.4% (*n* = 108) for the female sample, respectively. Taxometric analysis requires all standardized mean differences between the hypothetical categorical groups to be larger than Cohen’s *d* = 1.25. Furthermore, all indicators should correlate substantially with each other (mean *r* > 0.30), but the correlation should be substantially smaller within the hypothetical categorical groups (*r*_*wg*_ ≤ 0.30) (Ruscio et al., 2011).

## Results

### Taxometric Analyses of CU traits

#### Three-Indicator Sets

**Fig. 1:**
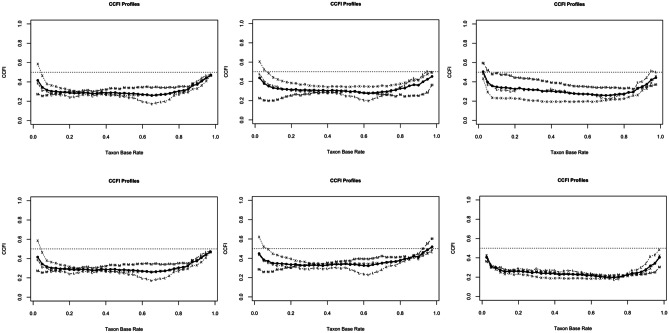
Results of the CCFI-profile analyses based on 3-indicator-sets (Essau et al., 2006↑ and Kimonis et al., 2015↓) for the total sample (left), male sample (middle), and female sample (right)

The overwhelming majority of all standardized mean differences exceeded the required cut-off of *d* = 1.25 (see Table 1). We observed an average correlation between *r* = 0.28 and *r* = 0.35 and smaller correlation coefficients in the hypothetical categorical groups (Essau et al., 2006: between *r* = 0.12 and *r* = 0.16 [taxon], between *r* = 0.24 and *r* = 0.29 [complement]; Kimonis et al. (2015): between *r* = 0.11 and *r* = 0.17 [taxon], between *r* = 0.25 and *r* = 0.30 [complement]). Figure 1 depicts the graphical taxometric results for the CCFI profile analyses of both three-indicator sets (Essau et al., 2006; Kimonis et al., 2015). Strong support for the superiority of a dimensional model was detected regarding the total sample (Essau et al.: CCFI mean profile = 0.316; Kimonis et al.: CCFI mean profile = 0.316), the male sample (CCFI mean profile = 0.328 / 0.376), and the female sample (CCFI mean profile = 0.322 / 0.248).

**Table 1:** Results from the Taxometric Analysis

Indicator selection	Sample	Base rate / Size of the potential taxon group	*d*	Indicator correlation	CCFI profile	Interpretation
**3-Indicators **(Essau et al., 2006)	Full sample	8.1% / *n* = 313	*M* = 1.16 Range = 0.86–1.64	Average: 0.31 Taxon: 0.12 Complement: 0.27	MAXEIG = 0.320 MAMBAC = 0.355 L-MODE = 0.289 *M* = 0.316	dimensional
	Male Sample	10.9% / *n* = 205	*M* = 1.03 Range = 0.70–1.64	Average: 0.28 Taxon: 0.12 Complement: 0.24	MAXEIG = 0.395 MAMBAC = 0.273 L-MODE = 0.325 M = 0.328	dimensional
	Female Sample	5.4% / *n* = 108	*M* = 1.32 Range = 1.07–1.50	Average: 0.34 Taxon: 0.16 Complement:0.29	MAXEIG = 0.245 MAMBAC = 0.425 L-MODE = 0.245 *M* = 0.322	dimensional
**3-Indicators (**Kimonis et al., 2015**)**	Full sample	8.1% / *n* = 313	*M* = 1.22 Range = 0.86–1.64	Average: 0.33 Taxon: 0.11 Complement: 0.28	MAXEIG = 0.320 MAMBAC = 0.355 L-MODE = 0.289 *M* = 0.316	dimensional
	Male Sample	10.9% / *n* = 205	*M* = 1.10 Range = 0.76–1.64	Average: 0.30 Taxon: 0.11 Complement: 0.25	MAXEIG = 0.397 MAMBAC = 0.412 L-MODE = 0.333 *M* = 0.376	dimensional
	Female Sample	5.4% / *n* = 108	*M* = 1.36 Range = 1.07–152	Average: 0.35 Taxon: 0.17 Complement: 0.30	MAXEIG = 0.228 MAMBAC = 0.261 L-MODE = 0.279 *M* = 0.248	dimensional
**4-Indicators (APSD)**	Full sample	8.1% / *n* = 313	*M* = 1.18 Range = 0.78–1.54	Average: 0.39 Taxon: 0.26 Complement: 0.34	MAXEIG = 0.221 MAMBAC = 0.341 L-MODE = 0.333 *M* = 0.292	dimensional
	Male Sample	10.9% / *n* = 205	*M* = 1.04 Range = 0.63–1.42	Average: 0.37 Taxon: 0.28 Complement: 0.32	MAXEIG = 0.268 MAMBAC = 0.360 L-MODE = 0.348 *M* = 0.313	dimensional
	Female Sample	5.4% / *n* = 108	*M* = 1.31 Range = 1.02–1.58	Average: 0.39 Taxon: 0.25 Complement: 0.35	MAXEIG = 0.206 MAMBAC = 0.496 L-MODE = 0.373 *M* = 0.359	dimensional
**4-Indicators** (Kliem et al., 2020)	Full sample	8.1% / *n* = 313	*M* = 1.24 Range = 0.86–1.54	Average: 0.39 Taxon: 0.25 Complement: 0.34	MAXEIG = 0.171 MAMBAC = 0.301 L-MODE = 0.399 *M* = 0.285	dimensional
	Male Sample	10.9% / *n* = 205	*M* = 1.12 Range = 0.76–1.42	Average: 0.38 Taxon: 0.28 Complement: 0.32	MAXEIG = 0.261 MAMBAC = 0.355 L-MODE = 0.380 *M* = 0.318	dimensional
	Female Sample	5.4% / *n* = 108	*M* = 1.37 Range = 1.07–1.58	Average: 0.39 Taxon: 0.22 Complement: 0.35	MAXEIG = 0.201 MAMBAC = 0.421 L-MODE = 0.365 *M* = 0.332	dimensional

Please note that in practice a final conclusion has to be drawn based on the mean CCFI profile instead of relying only on single CCFI profile values (i.e., MAXEIG, MAMBAC, or L-MODE; see Ruscio et al., 2018). Since the individual taxometric techniques provide independent evidence for the latent structure (see Ruscio et al., 2010), a certain variability of single CCFI profile values is to be expected

*d* Cohen’s, *CCFI profile* comparison curve fit index based on the CCFI profile method, *MAXEIG* maximum eigenvalue, *MAMBAC* mean above minus below a cut, *L-MODE* latent-mode factor analysis

#### Four-Indicator Sets

**Fig. 2:**
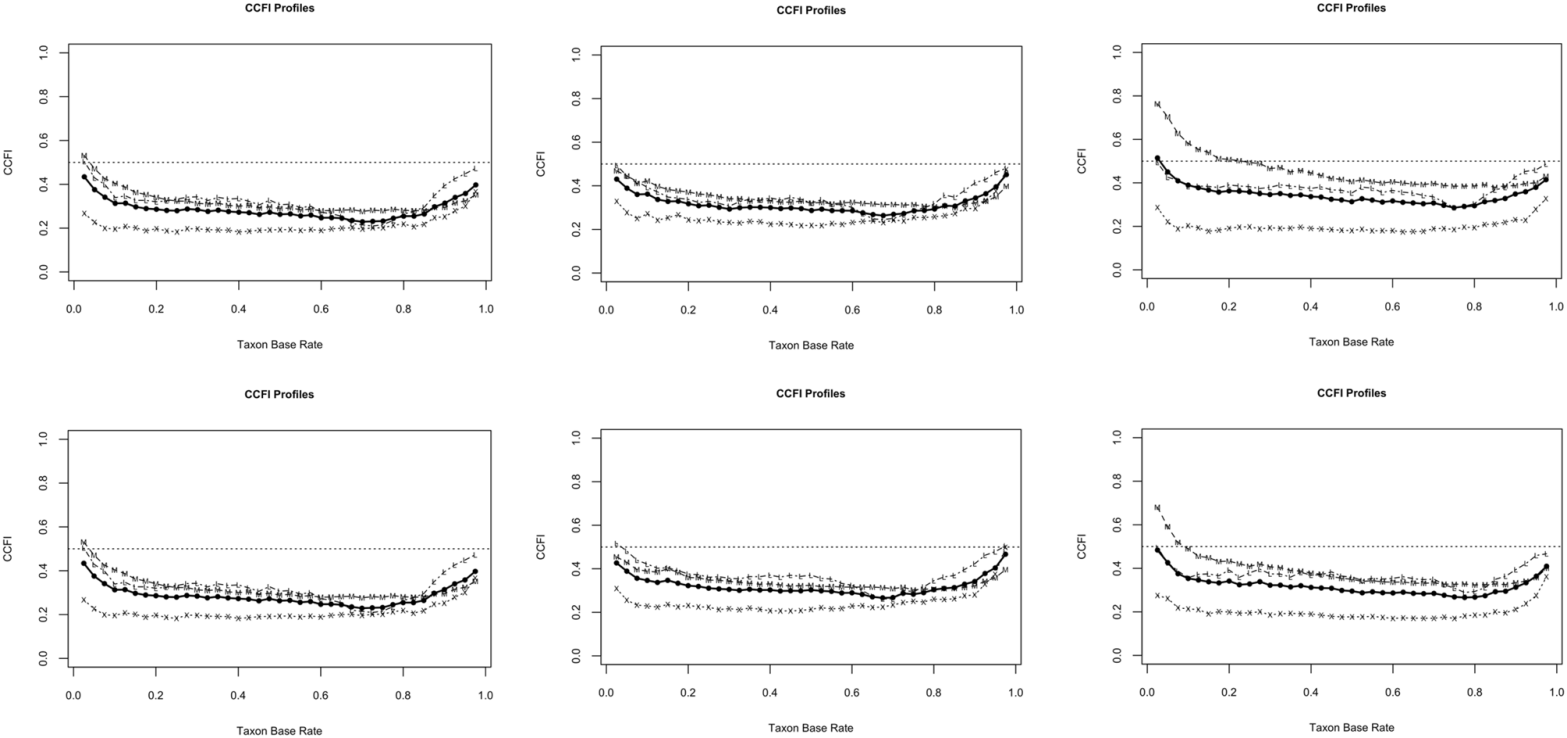
Results of the CCFI-profile analyses based on 4-indicator-sets (APSD↑and Kliem et al., 2020↓) for the total sample (right), male sample (middle), and female sample (left)

The majority of all standardized mean differences exceeded the required cut-off of *d* = 1.25 (see Table 1). We observed an average correlation between *r* = 0.39 and *r* = 0.39, and smaller correlations in the hypothetical categorical groups (APSD: between *r* = 0.25 and *r* = 0.28 [taxon], between *r* = 0.32 and *r* = 0.35 [complement]; Kliem et al., 2020: between *r* = 0.22 to 0.25 [taxon], between *r* = 0.32 and 0.35 [complement]). Figure 2 depicts the graphical taxometric results for the CCFI profile analyses of both four-indicator sets. Strong support for the superiority of a dimensional model was detected regarding the total sample (APSD: CCFI mean profile = 0.292; Kliem et al., 2020: CCFI mean profile = 0.285), the male sample (CCFI mean profile = 0.313 / 0.318), and the female sample (CCFI mean profile = 0.359 / 0.332).

## Discussion

The present study evaluated the latent nature of CU traits in a large sample of German ninth graders. Results of different indicator sets clearly suggested a dimensional solution. This finding is consistent with previous studies showing the dimensionality of psychopathy in adolescents (Edens et al., 2011; Murrie et al., 2007; Walters, 2014) as well as of ﻿early disruptive behavior in preschoolers (Kliem et al., 2018). However, further studies are necessary to substantiate this result in different samples (especially in samples of adolescents with Conduct Disorder) using different measurement approaches (e.g., teacher reports, parent reports). However, a dimensional structure of CU traits has important theoretical and practical implications: First, results indicate that the process of classifying individuals in dichotomous groups (CU + risk group) needs to be considered very carefully.

Second, it must be noted that people’s perceptions are affected when a construct is communicated as categorical (﻿e.g., Prentice & Miller, 2007). For example, the term “high-risk group” implies that the condition is more enduring than a dimensional construct. Therefore, the present analysis should give reason for researchers to avoid labeling individuals in order to decrease the associated risk of stigmatization in both scientific communication and therapeutic contexts. ﻿Our finding appears to be of particular importance in the context of CU traits, since this clinical picture is generally associated with a poor prognosis (e.g., Frick & White, 2008), a negative linguistic connotation with so-called “psychopathic traits” or “evil or dark personality” (Murrie et al., 2005), as well as treatment non-response (Falkenbach et al., 2003; Gretton et al., 2001; Hawes & Dadds, 2005; Kolk & Pardini, 2010; Masi et al., 2013; O’Neill et al., 2003; Spain et al., 2004; Waschbusch et al., 2007). Furthermore, labeling juveniles may also have a punishment-enhancing effect in legal settings, especially since the term ‘psychopath’ is associated with attributes such as cold-bloodedness, evilness, a pronounced lack of remorse and particularly high risk of recidivism (e.g., Berryessa, & Wohlstetter, 2019; Petrila & Skeem, 2003).

Third, for future research on CU traits, relevant implications can be drawn from the dimensionality of the construct. It seems particularly relevant that meaningful insights into the phenomenon can be derived from the study of subclinical samples. Furthermore, a dimensional structure suggests that a variety of risk factors affect the CU traits phenomenon (through addition and interaction). In this context, the polygenic nature of most psychiatric disorders should not be neglected, which are influenced by hundreds to thousands of genetic variations with very little (and interactive) effects (Moore et al., 2019).

### Limitations

There are many strengths of this study, including the very large and representative sample. However, the study has some limitations. Firstly, self-reports were the only data source used, so it is possible that the results are subject to monomethod bias (e.g., Kliem et al., 2015, 2016). When attempting to replicate our findings in future studies, investigators should ensure that other data sources are used, such as other self-report-measures, teacher/parent reports, clinical interviews, and/or observational measures. Secondly, data presented here is limited to the age group of ninth graders with a mean age of 15 years. Although the data are considered suitable for taxonomic analysis, within-group indicator correlations lie above the threshold of *r* = 0.30. According to Ruscio et al. (2006), difficulties in selecting appropriate indicators might itself be indirect evidence of dimensionality.^1^ According to Meehl (1995), a basis rate of ≥ 10% for the estimated taxon base rate (the proportion of taxon members in the sample) should be present. Our results fell below this value in some of the analyses. This is a limitation; however, it can also be pointed out that in the total sample the putative taxon group contains a very large number of cases (*N*_taxon_ > 300). It should be remembered that it is not only the base rate but also the absolute size of each group that determines the validity of a taxometric analysis. Furthermore, it may be noted that although the rate of ambiguous results for categorical data may increase slightly at base rates between 5 and 10%, erroneous results (e.g., an incorrectly determined solution) are rarely generated (see Ruscio et al., 2011). Thus, based on the findings of our study, which very clearly support a dimensional structure, there appears to be a relatively low risk that a dimensional solution was erroneously determined.

## Conclusion

In summary, the results of this study point to the need for critical reflection in defining a high-risk-group (CU +) in the context of CU traits. Although this classification may seem helpful to a clinician, it is possible that these classification systems impose clinical limitations that are not empirically defensible (see Haslam et al., 2006). With respect to the DSM-5 specifiers, the present results indicate that any classification into dichotomous groups needs to be considered very carefully. Furthermore, comparing prevalence rates across different groups (e.g., boys vs. girls, healthy vs. diseased, etc.) seems problematic. Indeed, the whole process of classifying individuals based on a sum score might be questionable (see Kliem et al., 2014).

## Footnotes

1 We have repeated the analyses with various indicator sets (i.e. forced three-, four and five-indicator sets using EFA-Promax/-Varimax, “best” (highest validity) items as indicators, “best” item pairs as indicators). All of these indicator sets showed similar properties regarding their amenability to the assumptions of the taxometric analysis. Irrespective of the indicator set analyzed, all results consistently pointed to a dimensional latent structure. Since, we regard the questionnaire used to be a valid operationalization of the construct at hand we consider these additional analyses to constitute sensitivity analyses corroborating our original conclusions.
